# Liver involvement in pediatric acute leukemia: subtype differences and complex hepatic injury as a novel marker for AML risk stratification

**DOI:** 10.3389/fonc.2026.1749226

**Published:** 2026-05-07

**Authors:** Yu-juan Xue, Yu Wang, Ai-dong Lu, Yue-ping Jia, Zhi-xiao Zhang, Lin Zhang, Xiaoyu Cao, Le-ping Zhang, Hui-min Zeng

**Affiliations:** Department of Pediatrics, Peking University People’s Hospital, Peking University, Beijing, China

**Keywords:** acute leukemia, children, liver involvement, prognosis, risk stratification

## Abstract

**Objectives:**

This study aimed to investigate the prevalence, pattern of liver involvement at diagnosis of pediatric acute leukemia, and its associations with clinical features and prognosis.

**Methods:**

From January 2016 to December 2017, 304 consecutive children newly diagnosed with acute leukemia (190 pre-B acute lymphoblastic leukemia (ALL), 25 T-ALL, and 89 acute myeloid leukemia (AML)), were enrolled. Liver involvement was defined as hepatomegaly (HM), hepatocellular injury (HI), hepatic dysfunction (HD) and cholestasis.

**Results:**

The prevalence of liver involvement varied significantly across leukemia subtypes: T-ALL (80.0%), pre-B-ALL (64.0%), and AML (37.0%) (P<0.001). HM was the most common isolated manifestation in all subtypes. Lower platelet counts were significantly associated with HM and HI in pre-B-ALL, and with HD in T-ALL (all P < 0.05). HI was positively correlated with age in pre-B-ALL (median age: 9.0 vs. 4.0 years, P < 0.001), while HM was associated with higher white blood cell (WBC) counts in T-ALL (228.0 vs. 10×10^9^/L, P = 0.035). HD showed a trend of association with elevated WBC counts in AML (P = 0.054). Liver involvement was not associated with early treatment response or long-term survival in ALL. In AML, however, complex hepatic injury and WBC count emerged as independent adverse prognostic factors: AML patients without liver involvement had the best 5-year overall survival (OS: 87.5 ± 4.4%) and event-free survival (EFS: 80.4 ± 5.3%), whereas those with complex hepatic injury had the poorest outcomes (5-year OS: 58.3 ± 14.2%, EFS:50.0 ± 14.4%).

**Conclusions:**

Liver involvement exhibits subtype-specific patterns in pediatric acute leukemia and is associated with age, WBC and platelet counts. Notably, complex hepatic injury may serve as a potential adverse prognostic marker for pediatric AML.

## Introduction

Acute leukemia, consisting of lymphoid (80%) and myeloid (20%) variants, is one of the most common malignancies in children, accounting for approximately 40% of all pediatric malignancies (1). While originating from bone marrow, extramedullary infiltration (especially of the liver) is a key feature of the disease (2). Leukemic liver involvement presents as hepatomegaly (HM), hepatocellular injury (HI), hepatic dysfunction (HD), or cholestasis, with asymptomatic HM being the most prevalent (3–6).

As the major metabolic organ for chemotherapeutic agents, liver function impairment may affect treatment efficacy and outcomes. However, previous studies on liver involvement in pediatric acute leukemia were limited by small sample sizes, lack of detailed subtype-specific analysis (e.g., separating pre-B-ALL from T-ALL), and inconsistent definitions of hepatic endpoints (3, 4). Particularly, the prognostic value of liver involvement in childhood AML remains insufficiently addressed. To fill these gaps, we conducted this large-cohort study to systematically explore the prevalence, pattern of liver involvement across leukemia subtypes, and its associations with clinical features and long-term outcomes.

## Materials and methods

### Patients

This single-center retrospective cohort study enrolled 313 consecutive children (0–18 years old) newly diagnosed with acute lymphoblastic leukemia (ALL) or acute myeloid leukemia (AML) at our institution between January 2016 and December 2017. Exclusion criteria were: (1) mixed-phenotype acute leukemia (n=3); (2) immediate treatment abandonment after diagnosis (n=6). Finally, 304 eligible patients were included, comprising 190 pre-B-ALL, 25 T-ALL, and 89 AML.

### Data collection

Patient data at initial diagnosis were reviewed and collected. General clinical records included patient demographic characteristics, physical examinations (presence of hepatosplenomegaly, central nervous system involvement), and disease subtypes. Blood tests records included white blood cell (WBC) count, platelet count, liver biochemistries (alanine aminotransferase (ALT), aspartate aminotransferase (AST), alkaline phosphatase (ALP), gamma glutamyl transferase (GGT)), serum total and conjugated bilirubin, serum albumin, international normalized ratio (INR), and viral serologies (Epstein-Barr virus, cytomegalovirus, herpes simplex virus, hepatitis A virus, hepatitis B virus, hepatitis C virus, and human immunodeficiency virus). No patient had serological findings suggesting acute viral hepatitis, moderate to severe malnutrition, or long-term exposure to hepatotoxic medications within 3 months before diagnosis. Imaging examinations records included abdominal ultrasound and computerized tomography performed at diagnosis or within a week of treatment where available. Early treatment response records, only for children with ALL, included the minimal residual disease (MRD) detected by multi-parameter flow cytometry after induction therapy.

### Treatment

The chemotherapy regimens administered to children with ALL and AML have been described in detail in our previous reports (7, 8).

### Definitions

In this study, the ALT, conjugated bilirubin, INR, and serum albumin levels at diagnosis were used as specific markers of liver involvement for the final analysis. The classification was established based on three core principles: ①alignment with international pediatric liver injury diagnostic standards; ② adaptation to the clinical context of pediatric acute leukemia; ③ high specificity and clinical accessibility to ensure reproducibility for future studies. The detailed definitions and rationale are as follows:

1. HM was assessed via two standardized, clinically accepted methods, with a unified protocol for all enrolled patients: ① Abdominal ultrasound (gold standard): Performed by board-certified pediatric ultrasound physicians, hepatomegaly was defined as the longitudinal diameter of the right liver lobe exceeding the age-matched upper limit of normal. A total of 300 patients (98.7% of the cohort) completed ultrasound examination at diagnosis. ② Standardized physical examination: Performed by attending physicians or above in the pediatric hematology department, hepatomegaly was defined as the lower edge of the liver palpable ≥2 cm below the right costal arch in the midclavicular line. Only 4 patients (1.3% of the cohort) were assessed solely via physical examination, all of whom had liver palpable ≥ 5 cm below the costal arch with clinically significant hepatosplenomegaly. Diagnostic consistency analysis showed excellent agreement between the two methods (Cohen’s Kappa = 0.82, 95% CI 0.76-0.88, P < 0.001) in patients who completed both assessments. Rationale: HM is the most direct, non-invasive marker of leukemic hepatic infiltration, and directly reflects structural liver invasion by leukemic cells, which is the core pathological basis of liver involvement in acute leukemia.
2. HI was defined as an ALT level above the age-matched upper limit of normal (ULN). Rationale: ALT was selected as the sole marker for hepatocellular injury for its superior liver specificity in the pediatric population: AST is widely expressed in extrahepatic tissues (myocardium, skeletal muscle, kidney), and its elevation is prone to be confounded by non-hepatic factors. This selection is fully consistent with pediatric hepatocellular injury diagnostic criteria recommended by North American Society for Pediatric Gastroenterology, Hepatology and Nutrition.
3. Cholestasis was defined as a conjugated bilirubin level above the age-matched ULN. ALP and GGT were not included in the core classification: ALP levels in children are strongly confounded by physiological bone growth with low liver specificity; no isolated GGT elevation was observed in our cohort, with all GGT elevations concurrent with elevated ALT or conjugated bilirubin, which would lead to redundant classification if included.
4. HD was defined as an INR above the ULN and a serum albumin level below the age-matched lower limit of normal (LLN) and no evidence of malnutrition/systematic inflammation. Rationale: This combined definition reflects the synthetic and metabolic functional impairment of the liver, which is more clinically meaningful for pediatric leukemia patients.
5. Complex hepatic injury was defined as the coexistence of hepatomegaly with at least one of the following abnormalities: hepatocellular injury or hepatic dysfunction, which indicates concurrent leukemic hepatic infiltration and parenchymal functional impairment. Isolated hepatomegaly, isolated biochemical abnormalities (HI or HD without hepatomegaly), and no liver involvement were defined as mutually exclusive subgroups for stratified analysis.
6. Complete remission (CR) was defined as < 5% leukemic blasts in marrow (mildly hypocellular to normal cellularity), normal peripheral blood counts, and the lack of clinical signs or symptoms attributable to the disease. The overall survival (OS) and event-free survival (EFS) were calculated from the date of diagnosis. The endpoint event for EFS was the first relapse or death in remission, whereas the endpoint event for OS was death from any cause. The final follow-up date was October 15, 2025.

### Statistical analysis

IBM SPSS 26.0 statistical software (SPSS Inc., Chicago, IL, USA) was used for the data analysis. Continuous variables were compared using the Mann–Whitney *U* test (for two groups) or Kruskal–Wallis *H* test (for three groups). Categorical variables were analyzed with the Pearson chi-squared test or Fisher’s exact test (when expected frequency < 5). Correlation between variables was assessed via Cohen’s kappa consistency test. OS and EFS were estimated using the Kaplan-Meier method, with log-rank test for comparisons. Multivariate Cox proportional hazards regression was performed to identify independent prognostic factors, with adjustment for age, white blood cell count, platelet count, INR level at diagnosis, cytogenetic risk stratification, liver involvement subgroups, central nervous system involvement and treatment protocols. Variable selection was performed as follows: variables with P < 0.1 in univariate Cox regression were included in the initial multivariate model, and forward stepwise regression (likelihood ratio test) was used for final variable screening, with an entry threshold of P < 0.05 and an exclusion threshold of P > 0.10. A two-sided P < 0.05 was considered statistically significant. The proportional hazards assumption was verified via global test. A total of 18 endpoint events (all-cause death) for OS and 25 endpoint events (relapse or death in remission) for EFS were included in the multivariate Cox model. The event-to-variable ratio was 6 for OS and 8.3 for EFS, which falls within the 5–10 EPV range widely recognized as acceptable for clinical research, especially in pediatric rare disease studies. All variables included in the model were predefined based on well-established prognostic factors for pediatric AML, ensuring the robustness and unbiasedness of the model results.

## Results

### Clinical characteristics

The clinical characteristics of the 304 enrolled children are presented in **Table 1**. The median age of patients in the AML group was 7 years, which was significantly higher than the 4 years old in the pre-B-ALL group (*P* < 0.001). Moreover, the proportion of children aged > 10 years was significantly higher in the AML group than in the pre-B-ALL group (41.6% vs. 21.1%; *P* < 0.001). In addition, the median WBC count in the T-ALL group was 136.4×10^9^/L, with 56% of children having a count greater than 100×10^9^/L, which were both significantly higher than those in the pre-B-ALL and AML groups (*P* < 0.001). In the AML group, 60.7% had a platelet count < 50×10^9^/L at initial diagnosis, which was significantly higher than 43.7% in the pre-B-ALL group (*P* < 0.001). There were no significant differences in other clinical characteristics between the three groups.

**Table 1:** Clinical features of the 304 enrolled children with leukemia.

Variables	Pre-B-ALL	T-ALL	AML
All, n (%)	190(100.0)	25(100.0)	89(100.0)
Sex			
Male	112(58.9)	19(76.0)	52(58.4)
Female	78(41.1)	6(24.0)	37(41.6)
*P*	0.100*	0.109**	0.934***
Age, range (y)	0.5-16.0	2.0-14.0	1.0-16.0
Age, median (y)	4.0	7.0	7.0
*P*	0.071*	0.405**	<0.001***
Age (y), n (%)			
<10	150(78.9)	17(68.0)	52(58.4)
≥10	40(21.1)	8(32.0)	37(41.6)
*P*	0.217*	0.387**	<0.001***
WBC, range (×10^9^/L)	1.0-619.1	1.1-691.9	0.5-418.0
WBC, median (×10^9^/L)	14.9	136.4	15.5
*P*	<0.001*	<0.001**	0.265***
WBC (×10^9^/L), n (%)			
<100	177(93.2)	11(44.0)	80(89.9)
≥100	13(6.8)	14(56.0)	9(10.1)
*P*	<0.001*	<0.001**	0.345***
Platelet, range (×10^9^/L)	3.0-390.0	15.0-563.0	2.0-1244.0
Platelet, median (×10^9^/L)	61.0	51.0	42.0
*P*	0.392*	0.153**	0.098***
Platelet(×10^9^/L), n (%)			
<50	83(43.7)	11(44.0)	54(60.7)
≥50	107(56.3)	14(56.0)	35(39.3)
*P*	0.975*	0.432**	<0.001***
CNSL, n (%)	12(6.3)	1(4.0)	11(12.4)
*P*	0.992*	0.404**	0.087***

*: Pre-B-ALL vs. T-ALL; **: T-ALL vs. AML; ***: Pre-B-ALL vs. AML.

ALL, acute lymphoblastic leukemia; AML, acute myeloid leukemia; WBC, white blood count; CNSL, central nervous system leukemia.

Cytogenetic and molecular data were available for all the AML patients, including karyotype analysis and fusion gene testing (*CBFβ-MYH11, RUNX1-RUNX1T1, PML-RARA, KMT2A* rearrangement, etc.). Among them, 41 pediatric patients presented with *CBF*-AML (*RUNX1-RUNX1T1* = 30, *CBFβ-MYH11* = 11), accounting for the highest proportion. Next were 7 pediatric patients with *KMT2A-r*. All patients received uniform chemotherapy according to the same protocol, with no treatment heterogeneity that would affect prognostic analysis.

### The prevalence and pattern of liver involvement in different childhood acute leukemia subtypes

HM was most common in the T-ALL group (80.0%), followed by the pre-B-ALL group (50.5%), and finally the AML group (25.8%), with statistically significant differences in pairwise comparisons among the three groups (**Table 2**). HI was found in 32.0% of the T-ALL group and 21.6% of the pre-B-ALL group, which was significantly higher than the 10.1% in the AML group (T-ALL vs. AML, *P* = 0.017; pre-B-ALL vs. AML, *P* = 0.020) (**Table 2**). HD was found in 20.0% of the patients in the T-ALL group, in 8.9% of those in the pre-B-ALL group and in 15.7% of those in the AML group; however, the differences between the groups were not statistically significant (**Table 2**). Cholestasis was least common in pre-B-ALL (1.6%), significantly lower than in T-ALL (8.0%) and AML (7.9%) (pre-B-ALL vs. T-ALL, *P* = 0.020; pre-B-ALL vs. AML, *P* = 0.020; **Table 2**), and thus omitted from further analysis due to its low prevalence.

**Table 2:** The prevalence of liver involvement in children with different types of acute leukemia.

Liver involvement	Pre-B-ALL	T-ALL	AML
All, n (%)	190(100.0)	25(100.0)	89(100.0)
Hepatomegaly, n (%)	96(50.5)	20(80.0)	23(25.8)
*P*	0.005*	<0.001**	<0.001***
Hepatocellular injury, n (%)	41(21.6)	8(32.0)	9(10.1)
*P*	0.243*	0.017**	0.020***
Hepatic dysfunction, n (%)	17(8.9)	5(20.0)	14(15.7)
*P*	0.173*	0.840**	0.093***
Conjugated hyperbilirubinemia (cholestasis), n (%)	3(1.6)	2(8.0)	7(7.9)
*P*	0.020*	1.000**	0.022***

*: Pre-B-ALL vs. T-ALL; **: T-ALL vs. AML; ***:Pre-B-ALL vs. AML.

ALL, acute lymphoblastic leukemia; AML, acute myeloid leukemia.

**Figure 1:**
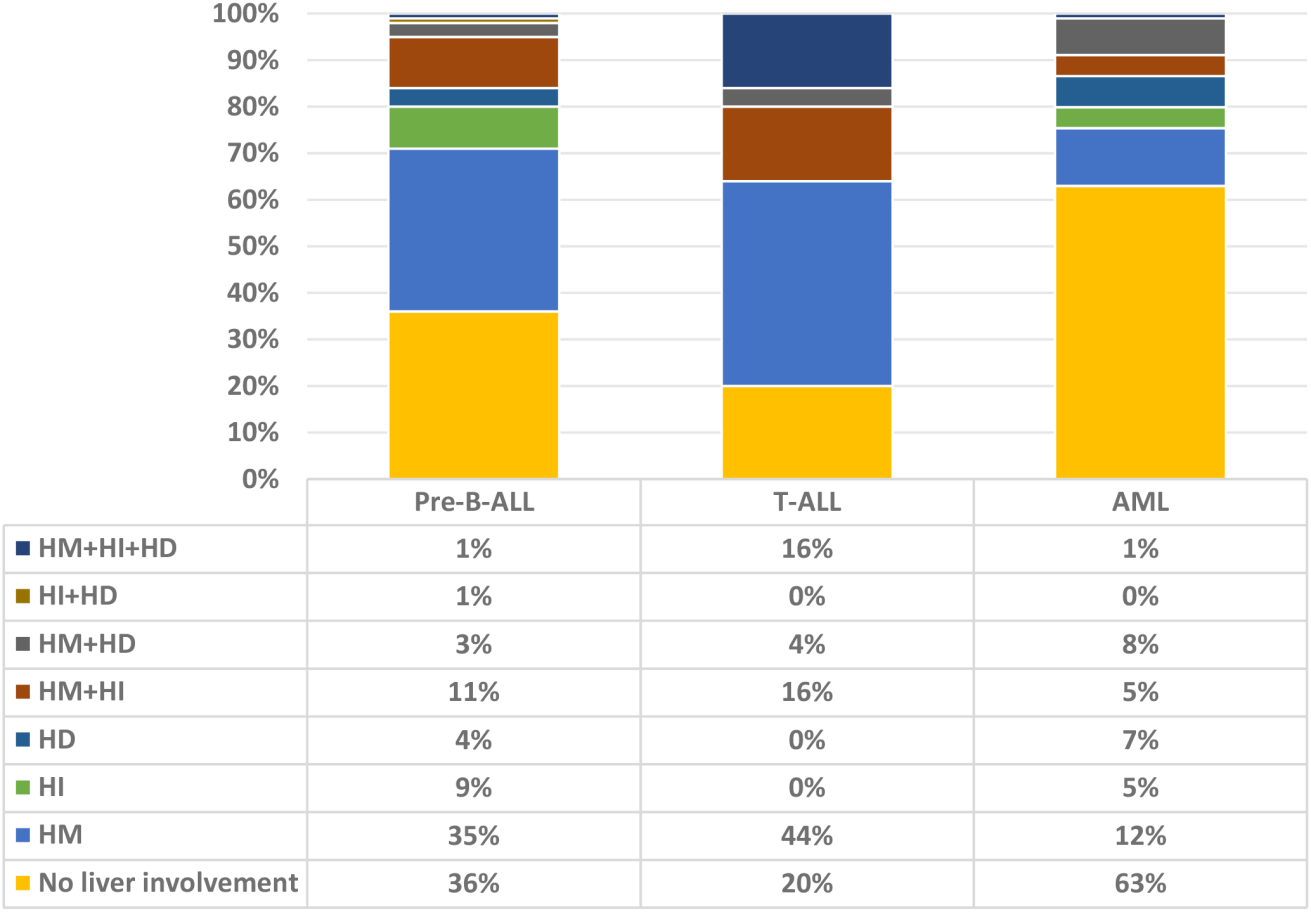
Distribution of patients with different liver involvement within each leukemia cohorts. ALL, acute lymphoblastic leukemia; AML, acute myeloid leukemia; HM, hepatomegaly; HI, hepatocellular injury; HD, hepatic dysfunction.

We further explored the association between HM, HI, and HD in each leukemia subtype and found that they were not strongly correlated with each other (**Supplementary Table 1**). The distribution of patients with different forms of liver involvement within each leukemia subtype is shown in **Figure 1**. The pattern of liver involvement varied greatly among the different leukemia subtypes (*P* < 0.001).

In pre-B-ALL, 36.0% of patients had no liver involvement, with isolated HM being the most common subtype (35.0%). HI occurred in ~10.0% of patients, with similar proportions of isolated HI and HI combined with HM. HD was uncommon and occurred usually in combination with other forms of liver involvement.

In children with T-ALL, the proportion with liver involvement was 80.0%, and the most common complication was HM alone (44.0%). Moreover, none of the patients had HD alone, HI alone, or a combination of both. HM, HI, and HD often occurred in combination.

Compared to those with pre-B-ALL and T-ALL, children with AML had the lowest incidence of liver involvement (37.0%). HM alone was the most common complication, accounting for approximately one-third of all cases. HM combined with other liver involvements accounted for a further one-third of the cases, and the remaining one-third of those with liver involvement were equally divided between HD alone and HI alone.

Splenomegaly was assessed via the same standardized protocol as hepatomegaly, and was significantly positively correlated with hepatomegaly in all three leukemia subtypes (Cohen’s Kappa = 0.79, 95% CI 0.72-0.86, *P* < 0.001).

### Association of age, WBC and platelet count with liver involvement at diagnosis

Given that age, WBC count and platelet count at presentation are well-established prognostic indicators in pediatric acute leukemia, we analyzed the associations between these three clinical factors and liver involvement phenotypes.

**Figure 2:**
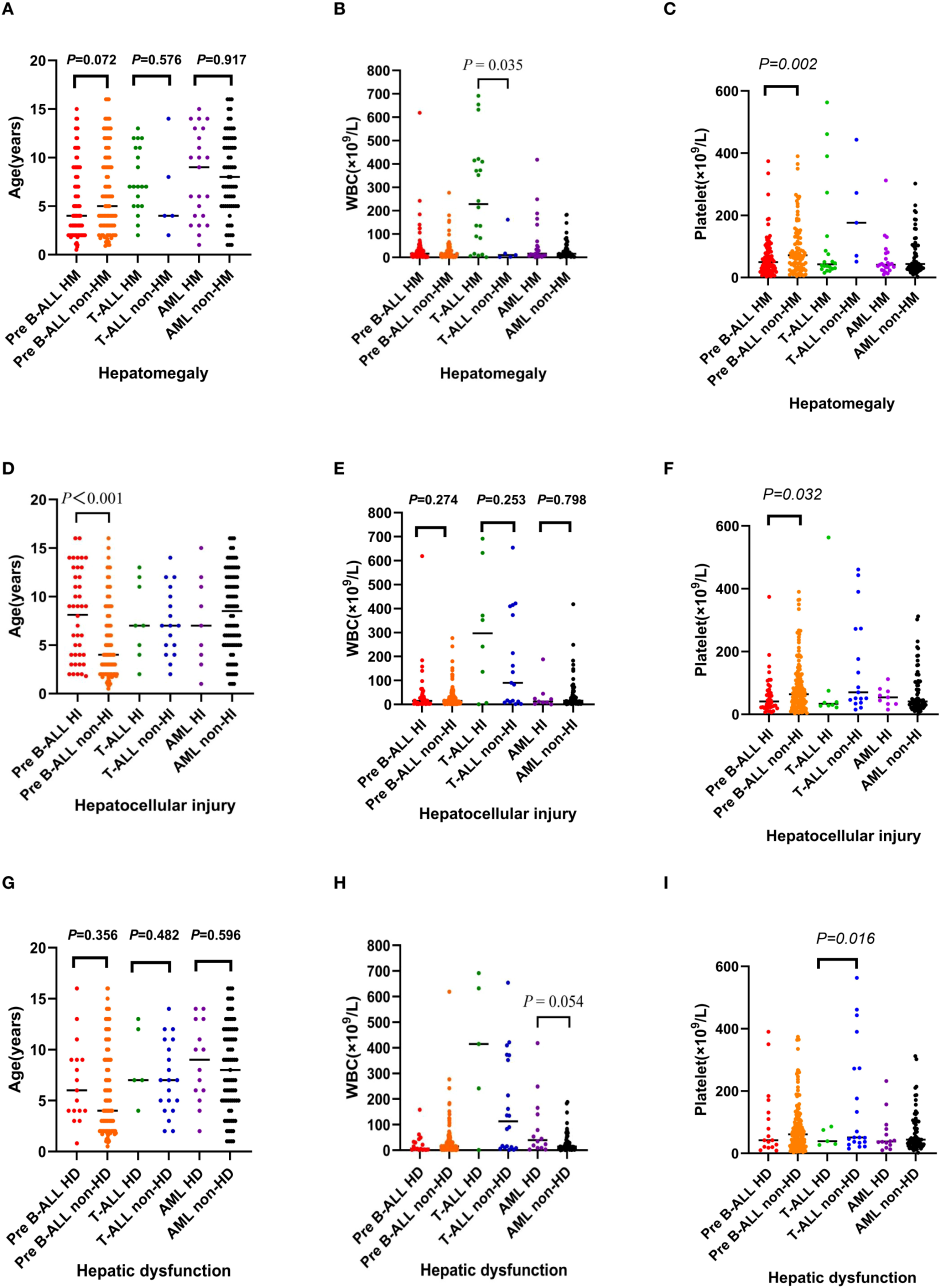
Comparison of initial age (years), white blood cell (WBC) and platelet count (×10^9^/L) between patients with and without liver involvement (hepatomegaly, hepatocellular injury, hepatic dysfunction) across different subtypes of pediatric acute leukemia. **(A-C)** hepatomegaly; **(D-F)** hepatocellular injury; **(G-I)** hepatic dysfunction. ALL, acute lymphoblastic leukemia; AML, acute myeloid leukemia; HM, hepatomegaly; HI, hepatocellular injury; HD, hepatic dysfunction; WBC, white blood cell count.

Age was not significantly associated with HM in any leukemia subtype (**Figure 2A**).

In the T-ALL group, the median WBC count at diagnosis for those with HM was 228.0×10^9^/L, which was significantly higher than the median of 10.0×10^9^/L in children without HM (*P* = 0.035) (**Figure 2B**).

In the pre-B-ALL group, the median platelet count at diagnosis for those with HM was 50.0×10^9^/L, which was significantly lower than the median of 72.0×10^9^/L in children without HM (*P* = 0.002) (**Figure 2C**).

In the pre-B-ALL group, the median age of children with HI (9.0 years) was significantly older than those without HI (4.0 years) (*P*<0.001) (**Figure 2D**).

In the AML group, children with HD tended to have a higher WBC count than those without HD (*P* = 0.054), however there was no significant association between WBC count and HI or HD in all leukemia subtypes (**Figures 2E, H**).

In the pre-B-ALL group, the median platelet count at diagnosis for those with HI was 41.0×10^9^/L, which was significantly lower than the median of 64.0×10^9^/L in children without HI (*P* = 0.032) (**Figure 2F**).

HD was not associated with age in patients with ALL or AML (**Figure 2G**).

In the T-ALL group, the median platelet count at diagnosis for those with HD was 39.0×10^9^/L, which was significantly lower than the median of 51.0×10^9^/L in children without HD (*P* = 0.016) (**Figure 2I**).

### Association of therapeutic efficacy with liver involvement at diagnosis

In terms of early treatment efficacy, we analyzed the relationship between liver involvement at diagnosis and MRD level after induction therapy in patients with ALL (**Supplementary Table 2**). The results showed no significant correlation between liver involvement and MRD level after induction chemotherapy, regardless of the MRD threshold of 0.1% or 0.01%.

**Figure 3:**
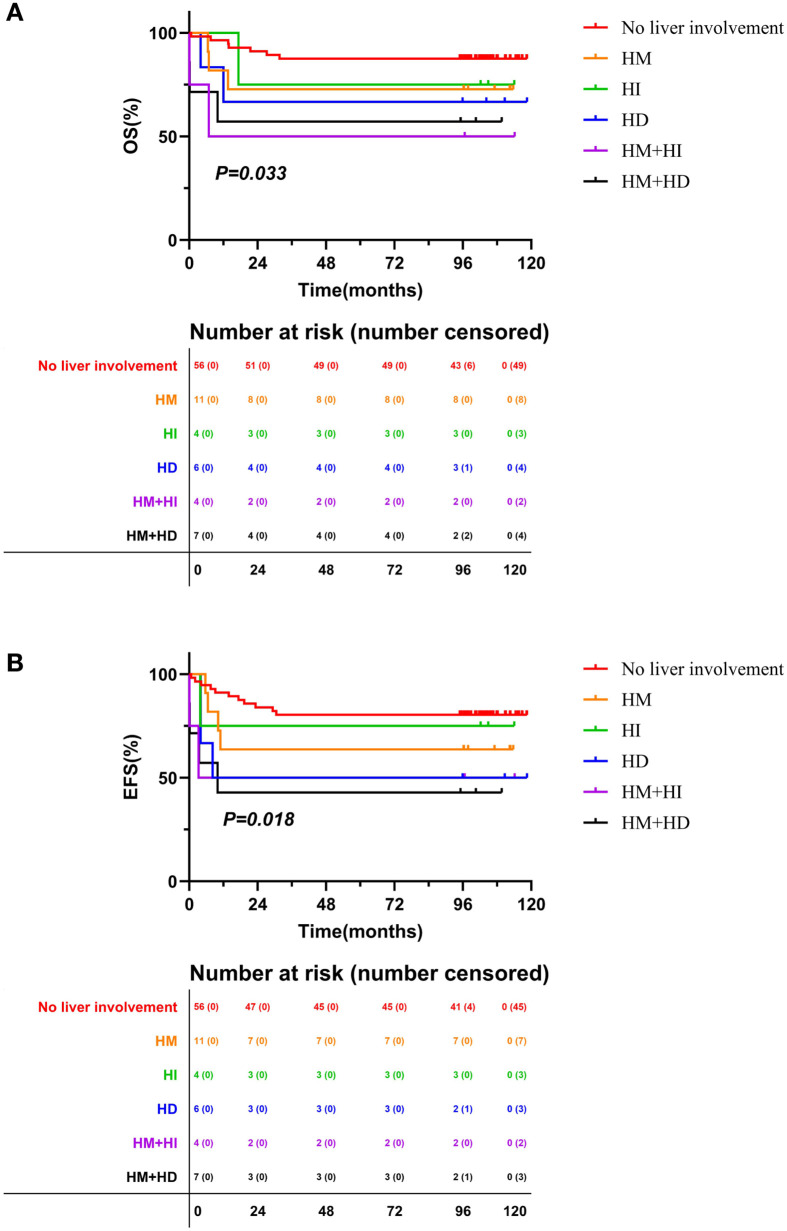
Overall survival and event free survival in AML patients with different liver involvement subgroups. **(A)** OS; **(B)** EFS; AML, acute myeloid leukemia; HM, hepatomegaly; HI, hepatocellular injury; HD, hepatic dysfunction; OS, overall survival; EFS, event free survival.

For long-term survival, we compared OS and EFS between the different liver involvement subgroups in children with ALL and AML (**Figure 3**; **Supplementary Figure 1**). Several subgroups were omitted from the survival analyses due to the small sample size, such as the HI+HD subgroup in ALL (n=1), the HM+HI+HD subgroup (n=1) and the HI+HD subgroup (n=0) in AML.

There were no significant differences in OS or EFS between the various liver involvement subgroups in patients with ALL (**Supplementary Figure 1**). In patients with AML, the types of liver involvement were significantly associated with OS and EFS (**Figure 3**). With a median follow-up time of 102.2 months, the 5-year OS and EFS of patients with AML was 79.8 ± 4.3% and 71.9 ± 4.8%, respectively. Patients with no liver involvement had the optimal survival, with 5-year OS and EFS of 87.5 ± 4.4% and 80.4 ± 5.3%, respectively. The worst survival was observed in patients with either HM+HI or HM+HD, with a 5-year OS of less than 60% and 5-year EFS of less than 50%. Patients with isolated hepatomegaly or isolated biochemical abnormalities (HI or HD without hepatomegaly) exhibited moderate survival rates, with a 5-year OS of approximately 72% and 5-year EFS of exceeding 60%.

Based on the subgroup survival outcomes described above, we further analyzed the survival outcomes of patients in isolated hepatomegaly group, isolated biochemical abnormalities group, no liver involvement group and complex hepatic injury group. Pairwise comparisons with Bonferroni correction (adjusted α=0.0083 for 6 comparisons) revealed that patients with complex hepatic injury had the poorest survival outcomes, with a 5-year OS of 58.3 ± 14.2% and 5-year EFS of 50.0 ± 14.4%, which was significantly lower than that of patients with no liver involvement (5-year OS: 87.5 ± 4.4%, EFS: 80.4 ± 5.3%, *P* = 0.004 for OS, *P* = 0.005 for EFS). There were no significant differences in OS or EFS between patients with isolated hepatomegaly, isolated biochemical abnormalities, and those with no liver involvement.

In AML, univariate and multivariate Cox regression confirmed complex hepatic injury (adjusted HR = 2.840, 95% CI:1.101-7.321, *P* = 0.031; **Supplementary Table 3**) and WBC count (adjusted HR = 3.076, 95% CI: 1.367-6.923, *P* = 0.007; **Supplementary Table 3**) as independent adverse prognostic factors for long-term survival. No serious adverse events related to forms of liver involvement were observed.

## Discussion

Our study systematically explored the subtype-specific patterns and prognostic value of liver involvement in pediatric acute leukemia, with findings that align with and extend previous research. Consistent with Sandart et al. (3), we observed a higher prevalence of liver involvement in lymphoid leukemia (T-ALL: 80.0%; pre-B-ALL: 64.0%) than in AML (37.0%), highlighting the distinct biological characteristics of different leukemia subtypes. Notably, our results further complement the adult AML study by Yilmaz et al. (9), which reported that liver dysfunction at diagnosis was associated with adverse risk stratification, MRD positivity, and poor prognosis. While Yilmaz et al. focused on adult patients and defined liver dysfunction based on multiple liver function test parameters, our pediatric cohort revealed that complex hepatic injury serves as a novel independent adverse prognostic factor for AML, with a hazard ratio of 2.840 (95% CI:1.101-7.321, *P* = 0.031). This consistency across age groups suggests that liver involvement may be a universal marker reflecting disease aggressiveness in AML, regardless of age. Notably, cholestasis was rare in pre-B-ALL (1.6%), but significantly more prevalent in T-ALL (8.0%) and AML (7.9%). Due to its extremely low overall prevalence, we omitted it from further prognostic and correlation analyses to avoid statistical bias caused by insufficient sample size. The clinical significance of cholestasis in pediatric acute leukemia warrants further exploration in larger cohorts with higher case numbers.

The subtype-specific associations between liver involvement and clinical factors (age, WBC, platelet count) further deepen our understanding of the underlying pathogenesis. In T-ALL, HM was significantly correlated with extremely high WBC counts (median: 228.0×10^9^/L in HM vs. 10.0×10^9^/L in non-HM, *P* = 0.035), which is consistent with the notion that high tumor burden promotes leukemic cell infiltration into the liver (10, 11). This is analogous to the adult AML study where liver dysfunction was linked to higher disease activity (9). In pre-B-ALL, hepatocellular injury (HI) was associated with older age (median: 9.0 years vs. 4.0 years, *P* < 0.001), which may be attributed to age-related differences in hepatic metabolism of leukemic toxins or immune responses to infiltration (4). For AML, hepatic dysfunction (HD) tended to correlate with elevated WBC counts (*P* = 0.054), echoing the positive association between tumor burden and liver impairment observed in both pediatric and adult studies (3, 9).

Notably, consistent with the hypothesis that platelet count reflects both baseline bone marrow involvement and systemic leukemic infiltration, we found significant subtype-specific correlations between platelet count and liver involvement phenotypes. In pre-B-ALL, lower platelet counts were significantly associated with the presence of HM and HI; in T-ALL, platelet count was significantly reduced in patients with HD. These findings align with previous pediatric leukemia studies showing that thrombocytopenia at diagnosis is a surrogate marker of higher tumor burden and more extensive extramedullary infiltration (12). These results collectively suggest that liver involvement in pediatric acute leukemia is not a random event but a direct reflection of disease biology, including tumor burden, bone marrow involvement, and subtype-specific infiltration propensity.

A key novel insight from our study is the differential prognostic value of liver involvement between ALL and AML. Unlike AML, where complex hepatic injury predicted poor 5-year overall survival and event-free survival compared to no liver involvement, liver involvement in ALL showed no correlation with early treatment response (MRD levels) or long-term survival. This discrepancy may be explained by several factors: first, AML and ALL differ in their extramedullary infiltration patterns—AML cells may exhibit more aggressive hepatic infiltration that impairs liver function and chemotherapeutic metabolism (9), whereas ALL-related liver involvement is often mild and reversible (3). Second, the chemotherapy regimens for AML are more hepatotoxic (8), and pre-existing liver impairment may exacerbate drug-induced liver injury, reducing treatment efficacy. Third, as suggested by Yilmaz et al. (9), liver dysfunction in AML may indicate systemic disease involvement beyond the bone marrow, which is inherently associated with worse prognosis. In contrast, ALL has a higher cure rate with more mature treatment protocols (13–16), and mild liver involvement may not be sufficient to affect outcomes.

Biologically, the prognostic specificity of our newly defined complex hepatic injury is well explained by the dual pathological features of leukemic liver involvement: hepatomegaly is the direct objective evidence of extensive leukemic cell infiltration in the liver, reflecting the stronger tissue invasion ability of tumor cells; while concurrent biochemical abnormalities indicate that the infiltration has caused irreversible parenchymal damage and impairment of hepatic synthetic and metabolic function. As the core metabolic organ for chemotherapeutic agents, pre-existing hepatic functional impairment may exacerbate drug-induced liver injury, reduce treatment efficacy, and ultimately lead to poor survival outcomes (9). By contrast, isolated hepatomegaly only reflects mild mechanical infiltration without functional damage, and isolated biochemical abnormalities are mostly mild, reversible hepatocellular injury without clear tumor infiltration evidence, thus having limited impact on long-term prognosis.

The definition of liver involvement in pediatric leukemia remains inconsistent across studies, which limits cross-study comparisons. We adopted a comprehensive definition incorporating HM (clinical/imaging), HI (ALT elevation), HD (INR elevation + hypoalbuminemia), and cholestasis (conjugated bilirubin elevation), which builds on the criteria used by previous studies (17, 18). Our observation that isolated HD is rare and typically combined with other liver involvement types highlights the necessity of integrating multiple biomarkers (rather than single enzymes) to assess liver function in pediatric leukemia. This is particularly relevant for AML, where HD may reflect more severe liver impairment and higher tumor burden (19). Future studies should aim to standardize liver involvement definitions, possibly incorporating both biochemical and imaging parameters, to enhance the reproducibility of prognostic findings.

From a clinical perspective, our findings provide a potential supplementary marker for the prognostic assessment of pediatric AML (20). Complex hepatic injury, defined as concurrent hepatomegaly and hepatic biochemical/functional impairment at diagnosis, can be easily assessed via routine physical examination, abdominal ultrasound, and standard liver biochemistry tests, which are widely available in clinical practice with no additional testing burden. However, given the retrospective and single-center nature of this study, these findings should be interpreted with caution. Complex hepatic injury may represent a potential prognostic marker for pediatric AML, which warrants further validation in larger, multicenter prospective cohorts before it can be incorporated into routine clinical risk stratification or guide treatment decision-making. For patients with complex hepatic injury, close monitoring of liver function during chemotherapy and proactive liver protection measures may help mitigate treatment-related toxicity.

In addition, liver function markers have expanded clinical utility beyond toxicity monitoring. They show promise for guiding individualized radiotherapy dosing to balance efficacy and hepatic safety (21, 22), and serve as early predictors of infectious complications in acute myeloid leukemia (23). These findings underscore the importance of routine liver function assessment in pediatric hematological malignancy management.

Our study has several limitations that warrant consideration. First, as a single-center retrospective study, selection bias may exist, and the generalizability of our findings is limited. The prognostic value of complex hepatic injury in pediatric AML requires further validation in larger, multicenter prospective cohorts before it can be applied to clinical practice. Second, the small sample size of some subgroups (e.g., HI+HD in ALL, n=1) limited the power of subgroup analyses. Third, we cannot completely exclude the residual confounding effect of subclinical systemic inflammation, mild leukemia-related coagulation abnormalities, or tumor consumption on liver function parameters, though strict enrollment criteria and necessary analysis have minimized this bias and confirmed the robustness of our core finding. Fourth, the primary endpoint of this study focused on hematological survival outcomes, and we did not systematically collect serial long-term follow-up data of chronic liver disease after chemotherapy completion. Finally, we did not explore the molecular mechanisms underlying the association between complex hepatic injury and AML aggressiveness, which warrants further investigation in future studies. Future studies could address these gaps by incorporating multi-omics analyses to identify molecular pathways linking liver involvement to AML aggressiveness, and by conducting long-term follow-up to evaluate both hematological and hepatic outcomes. Additionally, comparative studies between pediatric and adult AML patients may shed light on age-specific differences in liver involvement and its prognostic impact.

In conclusion, our large-cohort study demonstrates that liver involvement in pediatric acute leukemia exhibits distinct subtype-specific patterns and is associated with age and WBC count. Importantly, complex hepatic injury is a potential independent adverse prognostic marker for pediatric AML. These findings underscore the importance of comprehensive liver function assessment at diagnosis in pediatric leukemia, especially in AML. Further large-scale, multicenter prospective studies are warranted to validate the prognostic value of complex hepatic injury and its clinical application in risk stratification for pediatric AML.

## Data Availability

The original contributions presented in the study are included in the article/**Supplementary Material**. Further inquiries can be directed to the corresponding author.
