# Supplementary material for: Liver involvement in pediatric acute leukemia: subtype differences and complex hepatic injury as a novel marker for AML risk stratification
Source: Front Oncol. 2026 May 7;16:1749226. doi: 10.3389/fonc.2026.1749226 (PMC13189796; doi:10.3389/fonc.2026.1749226)
Supplement: Supplementary file 1 [file DataSheet1.docx]

**Supplemental Table 1 Correlation and P-values for correlation between hepatomegaly, hepatocellular injury and hepatic dysfunction in subtypes**

| Liver involvement | Pre-B-ALL  Kappa / *P* | 1. ALL   Kappa / *P* | AML  Kappa / *P* |
| --- | --- | --- | --- |
| Hepatomegaly vs. Hepatocellular injury | 0.048/ 0.42 | 0.211/ 0.086 | 0.196/ 0.032 |
| Hepatomegaly vs. Hepatic dysfunction | -0.012/ 0.764 | 0.118/ 0.211 | 0.294/ 0.040 |
| Hepatocellular injury vs. Hepatic dysfunction | -0.026/ 0.680 | 0.490/ 0.01 | -0.041/ 0.688 |

**ALL : acute lymphoblastic leukemia; AML : acute myeloid leukemia.**

| Liver involvement | n | MRD＜0.1%  n (%) | *P* | MRD＜0.01%  n (%) | *P* |
| --- | --- | --- | --- | --- | --- |
| NA | 3 |  |  |  |  |
| Hepatomegaly |  |  | 0.408 |  | 0.426 |
| Yes | 114 | 104(91.2) |  | 88(77.2) |  |
| No | 98 | 86(87.8) |  | 71(72.4) |  |
| Hepatocellular injury |  |  | 0.780 |  | 0.448 |
| Yes | 48 | 42(87.5) |  | 34(70.8) |  |
| No | 164 | 148(90.2) |  | 125(76.2) |  |
| Hepatic dysfunction |  |  | 0.873 |  | 0.194 |
| Yes | 22 | 19(86.4) |  | 19(86.4) |  |
| No | 190 | 171(90.0) |  | 140(73.7) |  |
| Any liver involvement |  |  | 0.467 |  | 0.315 |
| Yes | 140 | 127(90.7) |  | 108(77.1) |  |
| No | 72 | 63(87.5) |  | 51(70.8) |  |

**Supplemental Table 2 Relationship between liver involvement initially and MRD level after induction therapy in ALL patients**

**NA : not available; ALL : acute lymphoblastic leukemia; MRD: minimal residual disease.**

**A**

**
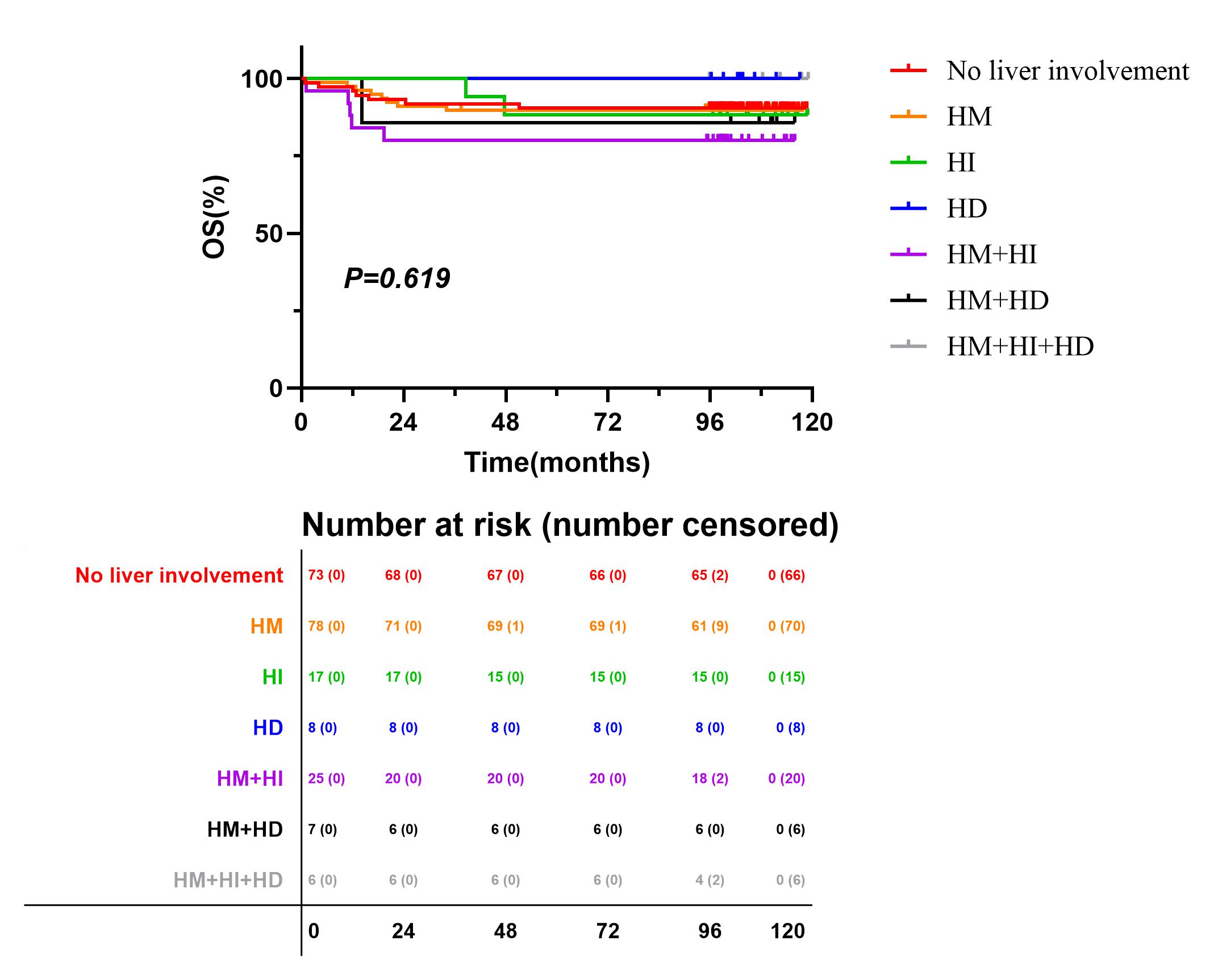
**

**B**

**
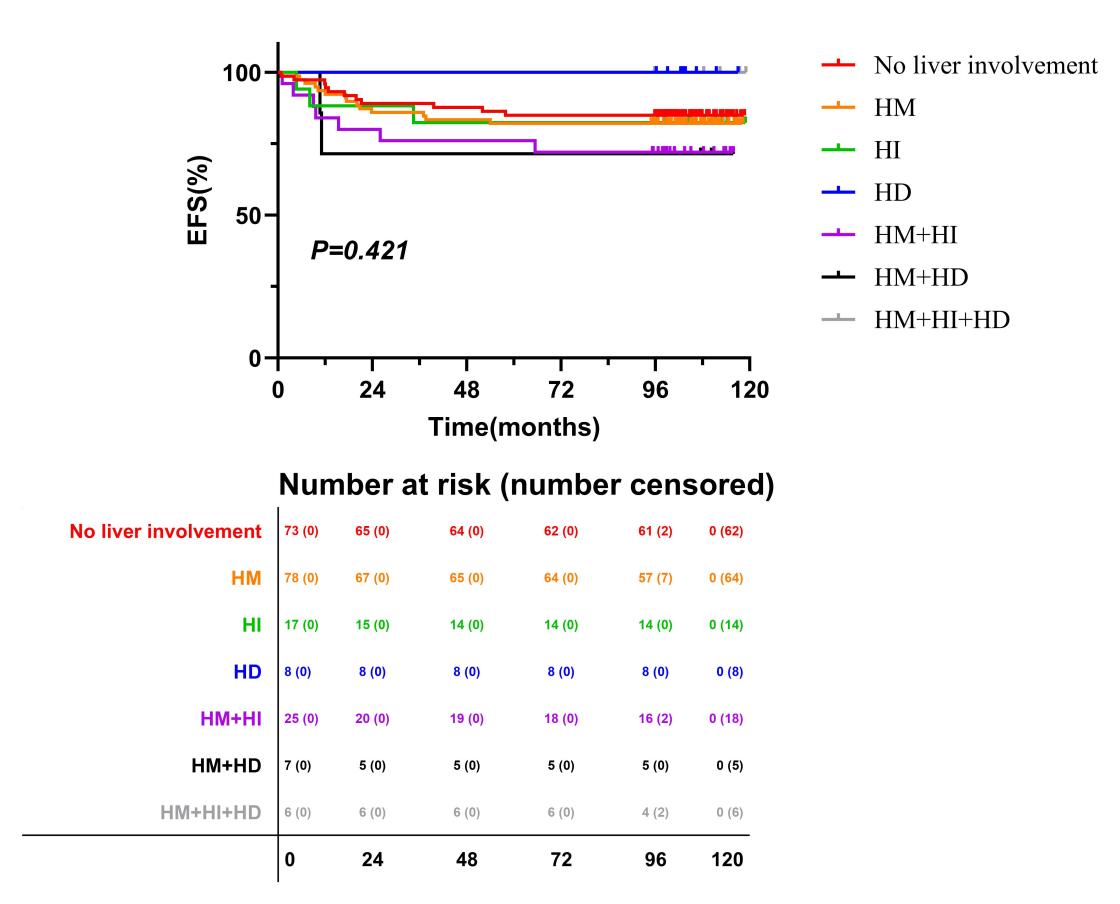
**

**Supplemental Figure 1. Overall survival and event free survival in ALL patients with different liver involvement subgroups. A : OS; B : EFS; ALL : acute lymphoblastic leukemia; HM : hepatomegaly; HI : hepatocellular injury; HD : hepatic dysfunction; OS : overall survival; EFS : event free survival.**

**Supplemental Table 3 Univariate and multivariate analysis of overall survival and event-free survival in AML group**

| Variable | OS | | EFS | |
| --- | --- | --- | --- | --- |
|  | Univariate(*P*) | Multivariate(*P*)  HR(95%CI) | Univariate(*P*) | Multivariate(*P*)  HR(95%CI) |
| Gender | 0.468 |  | 0.454 |  |
| Age (≥10) y | 0.840 |  | 0.266 |  |
| WBC≥50×10^9^/L | 0.025 | 0.058  2.531(0.969-6.609) | 0.004 | 0.007  3.076(1.367-6.923) |
| Platelet≥50×10^9^/L | 0.939 |  | 0.749 |  |
| INR level at diagnosis | 0.156 |  | 0.317 |  |
| CBF-AML | 0.082 | 0.212  0.509(0.176-1.471) | 0.095 | 0.233  0.592(0.250-1.402) |
| KMT2A-r AML | 0.506 |  | 0.289 |  |
| Non CR post-induction | 0.192 |  | 0.736 |  |
| Isolated hepatomegaly | 0.510 |  | 0.564 |  |
| Isolated biochemical abnormality | 0.422 |  | 0.308 |  |
| Complex hepatic injury | 0.014 | 0.062  2.748(0.950-7.949) | 0.014 | 0.031  2.840(1.101-7.321) |
| CNSL | 0.235 |  | 0.362 |  |
| HSCT | 0.879 |  | 0.976 |  |

**AML : acute myeloid leukemia; OS: overall survival; EFS: event free survival; WBC: white blood cell; CBF-AML: core binding factor-acute myeloid leukemia; CR: complete remission; CNSL: central nervous system leukemia; HSCT: hematopoietic stem cell transplantation.**
